# Synergistic use of gradient flipping and phase prediction for inline electron holography

**DOI:** 10.1038/s41598-022-17373-y

**Published:** 2022-08-02

**Authors:** Cigdem Ozsoy-Keskinbora, Wouter Van den Broek, Chris B. Boothroyd, Rafal E. Dunin-Borkowski, Peter A. van Aken, Christoph T. Koch

**Affiliations:** 1 Stuttgart Center for Electron Microscopy, Max Planck Institute for Solid State Research, Heisenbergstr. 1, 70569 Stuttgart, Germany; 2 Department of Physics, IRIS Adlershof, Humboldt-Universität Zu Berlin, 12489 Berlin, Germany; 3 Ernst Ruska-Centre for Microscopy and Spectroscopy with Electrons and Peter Grünberg Institute, Forschungszentrum Jülich, 52425 Jülich, Germany; 4 School of Materials Science and Engineering, Nanyang Technological University, Singapore, Singapore; 5 Present Address: Thermo Fisher Scientific, Eindhoven, The Netherlands

**Keywords:** Chemistry, Materials science, Optics and photonics, Physics

## Abstract

Inline holography in the transmission electron microscope is a versatile technique which provides real-space phase information that can be used for the correction of imaging aberrations, as well as for measuring electric and magnetic fields and strain distributions. It is able to recover high-spatial-frequency contributions of the phase effectively but suffers from the weak transfer of low-spatial-frequency information, as well as from incoherent scattering. Here, we combine gradient flipping and phase prediction in an iterative flux-preserving focal series reconstruction algorithm with incoherent background subtraction that gives extensive access to the missing low spatial frequencies. A procedure for optimizing the reconstruction parameters is presented, and results from Fe-filled C nanospheres, and MgO cubes are compared with phase images obtained using off-axis holography.

## Introduction

Fresnel fringes, the oscillatory features that appear at the edges of objects in images that have been recorded under (partially) coherent illumination and out-of-focus conditions, are commonly used in transmission electron microscopy (TEM) for detecting materials that consist of light elements and as a guide for focusing. They contain information about the phase of the complex exit wave that is encoded in a recorded image as well. Fresnel fringes are sensitive to very small relative optical path differences (phase shifts) imparted on an electron when it passes through a sample. Their analysis allows the retrieval of information about fine phase differences.

The retrieval of an electron wave function in the TEM by means of interference between a reference wave and an object wave was first proposed and demonstrated by Gabor^1^, who termed the technique ‘holography.’ Electron holography is currently applied in a wide variety of investigations^2–9^, which rely on the sample-imposed phase shift in an electron wave function. After the development of the laser, Leith and Upatniks^10^ developed an off-axis configuration that helped to overcome the twin-image problem^11^ that affects the interpretation of single exposures in inline holography. Möllenstedt^12^ showed that, for electrons, a charged wire could work effectively as an optical biprism, thereby allowing an off-axis reference wave to be generated in the TEM. Once highly coherent electron sources were developed by Tonomura^13^, off-axis electron holography, where the spatial resolution that can be achieved is determined in part by the interference fringe spacing^4^, became a routine technique. The requirement for a high biprism voltage, excellent microscope stability, high source brightness, and long acquisition times renders high spatial frequency imaging challenging for off-axis electron holography.

Inline electron holography is based on the interference of the electron wave function transmitting the object with itself. One way to overcome the twin image problem is to record a series of images at several planes of focus, typically above and below to the in-focus plane. If the defocus is small, then interference is local, and fine Fresnel fringes carry information about high spatial frequencies in the phase. At larger defocus values, partial spatial coherence damps the contrast of Fresnel fringes because the lateral coherence length imposes an upper limit on the distance across which interference occurs. Inline electron holography, e.g., using iterative reconstruction algorithms based on initial developments by Gerchberg and Saxton^14^ and Misell^15^, is therefore efficient for recovering high spatial frequency phase information but less so for lower spatial frequencies, where even under noise- and incoherent scattering-free conditions they require very many iterations and substantial computing power to converge^16^. To a degree, phase changes across distances larger than the lateral coherence length can be obtained by using non-interferometric reconstruction algorithms such as the transport of intensity equation (TIE)^17^. A combination of both approaches can then give access to high and low spatial frequencies in the phase^18^. However, in addition to amplifying low-frequency noise, the TIE is based on an elliptic partial differential equation of second order. Its solution suffers from a lack of knowledge of boundary conditions at the edges of the field of view.

Several iterative reconstruction algorithms have been developed based on linear and non-linear imaging models, i.e., whether the image contrast is considered to be due to interference between an undiffracted (reference) wave and diffracted waves or whether full interference between all partial waves transmitted by the object is taken into account. Although each algorithm has its strengths, none of them are able to recover the complete spectrum of spatial frequencies of the phase. Initializing inline reconstruction with the phase reconstructed from an off-axis electron hologram allows the phase to be recovered at all spatial frequencies, but this approach requires a biprism to be installed in the TEM^19^.

While off-axis and inline electron holography reconstructions have been compared before^20–22^, the purpose of this communication is to introduce an inline electron holography reconstruction algorithm that allows a wide range of spatial frequencies to be recovered by combining gradient flipping^23,24^ and phase prediction with incoherent background subtraction and a flux-preserving non-linear imaging model^25,26^.

## Experimental details

Gradient-flipping and phase prediction assisted flux-preserving full-resolution wave reconstruction (GPFRWR) inline electron holography, conventional iterative inline electron holography, and off-axis electron holography experiments were carried out for two different samples: Core–shell Fe-filled C nanospheres and MgO cubes.

Off-axis electron holograms and defocus series of bright-field TEM images of core-shell Fe-filled C nanospheres and MgO cubes (See in supplementary information (SI)) were recorded at an accelerating voltage of 300 kV using an FEI Titan 80–300 TEM equipped with an electron biprism and a Gatan imaging filter with a 2048 × 2048 pixel charge-coupled device camera. A 10 eV energy-selecting slit was inserted and centered on the zero-loss peak for both off-axis and inline electron holography in order to reduce the contribution of inelastically scattered electrons.

For off-axis electron holography, the biprism voltage was set to 139 V (0.53 nm fringe spacing) for the Fe-filled C nanospheres and 80.5 V (0.45 nm fringe spacing) for the MgO cubes. Holografree software^27^ was used for off-axis electron hologram reconstruction.

For inline electron holography, focal series were recorded using the FWRWtools^28^ plugin for Digital Micrograph, which automates image acquisition and compensates for specimen drift. The nominal defocus values were set according to the formula

1 $$\Delta {f}_{n}={\Delta }_{0}\frac{{\left|n\right|}^{p}n}{\left|n\right|},$$

with *Δ *_*0*_= defocus step and *n* = … − 2, − 1, 0, 1, 2 …. If *p* = 2 or *p* = 3, phase information can be sampled very efficiently for both low and high spatial frequencies^26^. For the Fe-filled C nanospheres, the defocus values spanned the range − 3.6 to 3.6 µm, with 600 nm defocus steps and linear increments (*p* = 1). For the MgO cubes, the defocus values spanned the range − 260 to 330 nm, with 40 nm defocus steps and linear increments (*p* = 1).

Exit surface wave functions were reconstructed using the flux-preserving imaging model and corresponding reconstruction algorithm^25^, combined with gradient flipping and phase prediction. Details of the reconstruction procedure are given in the theory section.

In order to monitor convergence of the reconstruction, a mismatch or residual *M* value was computed between images simulated from the reconstructed wave function and the experimental data^29^, according to the expression

2 $$M=\frac{\sum_{i,j}\left|{I}_{{sim}_{i,j}}-{I}_{{exp}_{i,j}}\right|}{\sum_{i,j}{I}_{{exp}_{i,j}}},$$

where *I*_*sim*_ and *I*_*exp*_ are the reconstructed and recorded image intensities, respectively, and the indices *i* and *j* run over the first and second dimension of the image.

## Theory

The motivation for the development of gradient-flipping and phase prediction assisted flux-preserving full-resolution wave reconstruction (GPFRWR) is that, in most TEM investigations of specimens that do not generate magnetic and electrostatic fringing fields, the phase in vacuum regions within the field of view has a constant value. The slab geometry of many TEM samples may also result in approximately flat regions of phase in large parts of the specimen, at least at medium spatial resolution. In other words, the gradient of the phase is often quite sparse, especially when excluding high spatial frequencies.

In its original application, the charge flipping algorithm for solving crystal structures from X-ray diffraction data^30^ is very effective at finding a sparse solution in the charge density domain by flipping the signs of small values while retaining values above a given threshold and enforcing consistency with measured diffraction intensities. After demonstrating its feasibility for removing low spatial frequency noise in TIE reconstructions^23,24^, we adapted the principle to non-linear inline electron holography by inserting a phase-modifying procedure every few iterations (e.g., every third iteration) in an iterative reconstruction algorithm (the FRWR algorithm^25,26^), flipping the signs of small values of each of the two components of the gradient of the phase and reducing their amplitudes, yielding a modified gradient ${\overrightarrow{G}}^{^{\prime}}\left(\overrightarrow{r}\right)$. This procedure was implemented simply by multiplying these values by a scaling factor β that was slightly larger than − 1 (e.g., β = − 0.97). This operation was only performed within the field of view defined by the experimental data. The size of the array defining the reconstructed phase was larger than the field of view, in order to be able to accommodate non-periodic boundary conditions. Whereas the larger array has periodic boundary conditions, the array corresponding to the field of view of the experimental data can have any boundary because it lies within the typically 1.5 to 2 times wider reconstruction array that has periodic boundary conditions^16^. Once the small gradients have been flipped, the modified phase is obtained by integrating the modified gradient ${\overrightarrow{G}}^{^{\prime}}\left(\overrightarrow{r}\right)$ The Fourier transform of the modified phase ${\phi }^{^{\prime}}\left(\overrightarrow{q}\right)$ is obtained from ${\overrightarrow{G}}^{^{\prime}}\left(\overrightarrow{r}\right)$ by the following operation:

3 $${\phi }^{^{\prime}}\left(\overrightarrow{q}\right)=\phi \left(\overrightarrow{q}\right)\left[1-\mathit{exp}\left(-{r}_{c}^{2}{q}^{2}\right)\right]+FT\left[\overrightarrow{\nabla }\cdot {\overrightarrow{G}}^{^{\prime}}\left(\overrightarrow{r}\right)\right]\frac{\mathit{exp}\left(-{r}_{c}^{2}{q}^{2}\right)}{-{q}^{2}},$$

where *r*_c_ is the length scale below which the flipping of the gradient has very little effect, i.e. this value can be chosen to apply gradient flipping only to spatial frequencies for which the iterative reconstruction algorithm would otherwise converge only very slowly. Dividing by – q^2^ effectively implements an inverse Laplace operator. At *q* = 0, we multiply by 0 instead of dividing by q^2^. This approach can be justified by the argument that the absolute phase is not a well-defined physical quantity. Multiplication by 0 at *q* = 0 will cause the mean of the reconstructed phase to be set to 0. After reconstruction, an offset that corresponds to the mean of the phase-in vacuum can be subtracted. This subtraction was applied to the phase maps shown in this paper.

Using the above expression, gradient flipping affects mostly spatial frequencies in the phase that are larger than *r*_*c*_ by using a Gaussian taper to transition between the ranges *r* > *r*_*c*_ and *r* < *r*_*c*_. Since the iterative part of the focal series reconstruction algorithm reconstructs primarily high spatial frequencies in the phase and requires many iterations to have an effect on lower spatial frequencies^16^, Eq. (3) ensures that gradient flipping does not affect the convergence of the iterative reconstruction algorithm significantly. It may even help to speed up convergence, especially in regions where large areas of the phase are flat (*e.g.*, for nanoparticles on homogeneous supports or if the field of view contains large areas of empty space).

In order to reduce the detrimental effect of incoherent scattering (e.g., electron–phonon and electron-plasmon interactions or the excitation of core electrons) on the interpretability of experimental TEM images using elastic scattering theory, our algorithm is based on the following flux-preserving imaging model, which is modified to include an incoherent background:

4 $${I}_{\Delta f}\left(\overrightarrow{r}\right)=\left[{\left|{\Psi }_{\Delta f}\left(\overrightarrow{r}\right)\right|}^{2}+{I}_{incoherent}\left(\overrightarrow{r}\right)\right]\otimes {E}_{s,\Delta f}\left(\overrightarrow{r}\right),$$

where *Ψ*_*Δf*_ (*r*) is the coherent electron wave function defocused by an amount Δ*f* in the plane of the detector, *E*_*s, Δf*_(*r*) is the inverse Fourier transform of the spatial coherence envelope^15^ and *I*_*incoherent*_ (*r*) is the intensity distribution of the incoherent background that is determined in addition to the electron wave function *Ψ*_*0*_ (*r*) at the exit surface of the specimen. Since only a single incoherent image is assumed, this residual image is taken to be the same for all images in the focal series. The incoherent intensity is strictly positive. At each iteration, after the average of the remaining difference between the forward simulated image and the experimental image has been added to the incoherent intensity array, this array is multiplied by a damping factor, which is set to 0.98 by default but can be changed when calling the reconstruction program. Thus, only a fraction of the minimum observed difference between simulated and experimental images in each pixel is assigned to this incoherent intensity array, ensuring that only image counts that cannot be attributed to the coherent imaging process are considered for assignment to the incoherent background.

The FRWR algorithm^25,26^ accelerates the retrieval of low spatial frequency phase information by using a ‘phase prediction’ mechanism. In addition to directly minimizing the difference between simulated and experimental images in a manner that is somewhat similar to conventional Gerchberg-Saxton-type focal series reconstruction algorithms^25^, the FRWR algorithm also explicitly updates the phase. This is done by first computing a phase update that is motivated by the transport of intensity equation (TIE) as follows:

5 $$\begin{array}{c}\Delta \varphi \left(\overrightarrow{r}\right)={\varphi }^{exp}\left(\overrightarrow{r}\right)-{\varphi }^{sim}\left(\overrightarrow{r}\right)\\ =\frac{2\pi }{\lambda }\frac{{\nabla }^{-1}{I}_{0}^{-1}\left(\overrightarrow{r}\right){\nabla }^{-1}\left({I}_{+\Delta f}^{\mathrm{exp}}\left(\overrightarrow{r}\right)-{I}_{-\Delta f}^{\mathrm{exp}}\left(\overrightarrow{r}\right)\right)}{2\Delta f}-\frac{2\pi }{\lambda }\frac{{\nabla }^{-1}{I}_{0}^{-1}\left(\overrightarrow{r}\right){\nabla }^{-1}\left({I}_{+\Delta f}^{sim}\left(\overrightarrow{r}\right)-{I}_{-\Delta f}^{sim}\left(\overrightarrow{r}\right)\right)}{2\Delta f}\\ =\frac{2\pi }{\lambda }\frac{{\nabla }^{-1}{I}_{0}^{-1}\left(\overrightarrow{r}\right){\nabla }^{-1}\left({I}_{+\Delta f}^{\mathrm{exp}}\left(\overrightarrow{r}\right)-{I}_{+\Delta f}^{sim}\left(\overrightarrow{r}\right)\right)}{2\Delta f}-\frac{2\pi }{\lambda }\frac{{\nabla }^{-1}{I}_{0}^{-1}\left(\overrightarrow{r}\right){\nabla }^{-1}\left({I}_{-\Delta f}^{\mathrm{exp}}\left(\overrightarrow{r}\right)-{I}_{-\Delta f}^{sim}\left(\overrightarrow{r}\right)\right)}{2\Delta f}\end{array}$$

*i.e.*, a TIE-like update of the phase that takes into account all *N* planes of focus can be computed using the expression

6 $$\Delta \varphi (\overrightarrow{r})=\frac{2\pi }{N\lambda }{\sum }_{n=1}^{N}\frac{{\nabla }^{-1}{I}_{0}^{-1}(\overrightarrow{r}){\nabla }^{-1}\left({I}_{\Delta {f}_{n}}^{exp}(\overrightarrow{r})-{I}_{\Delta {f}_{n}}^{sim}(\overrightarrow{r})\right)}{\Delta {f}_{n}}.$$

Before adding this phase update to the phase of the current estimate of the retrieved wave function, its magnitude is limited to a maximum value *ϕ*_max_, the so-called phase prediction threshold (PPT), by using exponential compression for high values of Δ*ϕ* (*r*) to avoid a sharp threshold:

$$\varphi \left(\overrightarrow{r}\right)= \left\{\begin{array}{ll}sign\left(\varphi \left(\overrightarrow{r}\right)\right)\frac{{\varphi }_{max}}{2}\left[2-exp\left(\frac{\frac{{\varphi }_{max}}{2}-\left|\varphi \left(\overrightarrow{r}\right)\right|}{\frac{{\varphi }_{max}}{2}}\right)\right]& if \left|\varphi \left(\overrightarrow{r}\right)\right|\ge \frac{{\varphi }_{max}}{2}\\ \varphi \left(\overrightarrow{r}\right)& if \left|\varphi \left(\overrightarrow{r}\right)\right|< \frac{{\varphi }_{max}}{2}\end{array}\right.$$

The use of this limit is important because incoherent contributions to the signal and noise prevent a perfect match from being obtained between experimental and simulated intensities, causing the expression in parentheses in Eq. (6) to practically never reach zero. The continual addition of a non-zero update to the phase would therefore result in diverging behavior of the reconstruction algorithm.

## Results and discussion

**Figure 1:**
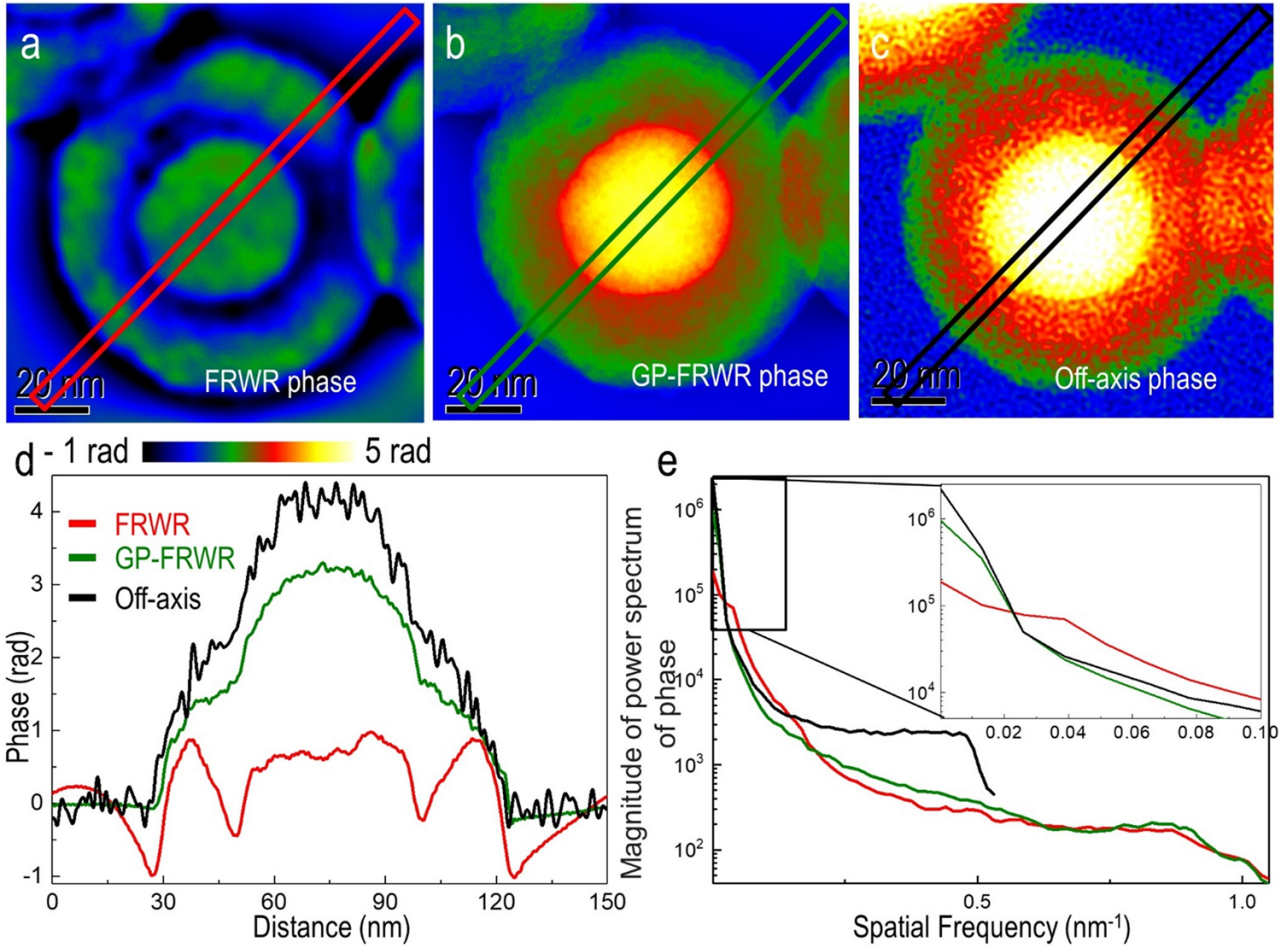
Phase images of Fe-filled C nanospheres were reconstructed using (**a**) FRWR**,** (**b**) GPFRWR, and (**c**) off-axis electron holography. (**d**) Line profiles extracted from the three phase reconstructions. (**e**) Radially-averaged power spectra of the three phase images.

Figure 1 shows the results obtained from Fe-filled C nanospheres. Phase images obtained using conventional inline (FRWR) reconstruction, GPFRWR, and off-axis electron holography are shown in Fig. 1a–c, respectively. Conventional FRWR recovered the phase with a range of approx. 2 radians, whereas the off-axis reconstruction shows that the phase spans a range of 4.12 ± 0.144 radians. The discrepancy is reduced to ~ 80% of the off-axis phase when GPFRWR is applied. The large phase variations at the edges, which are associated with missing low spatial frequencies, are prominent in the conventional FRWR reconstruction in Fig. 1d (red line), but are significantly reduced when using gradient flipping and phase prediction combined. In Fig. 1e, power spectra are shown for the three reconstruction schemes, confirming that low spatial frequency information obtained using the GPFRWR algorithm is much closer to that obtained using off-axis holography than FRWR. The remaining discrepancies in the phase retrieved by off-axis holography and the GPFRWR reconstruction are likely in large part due to the perfect energy filtering of off-axis holography.

**Figure 2:**
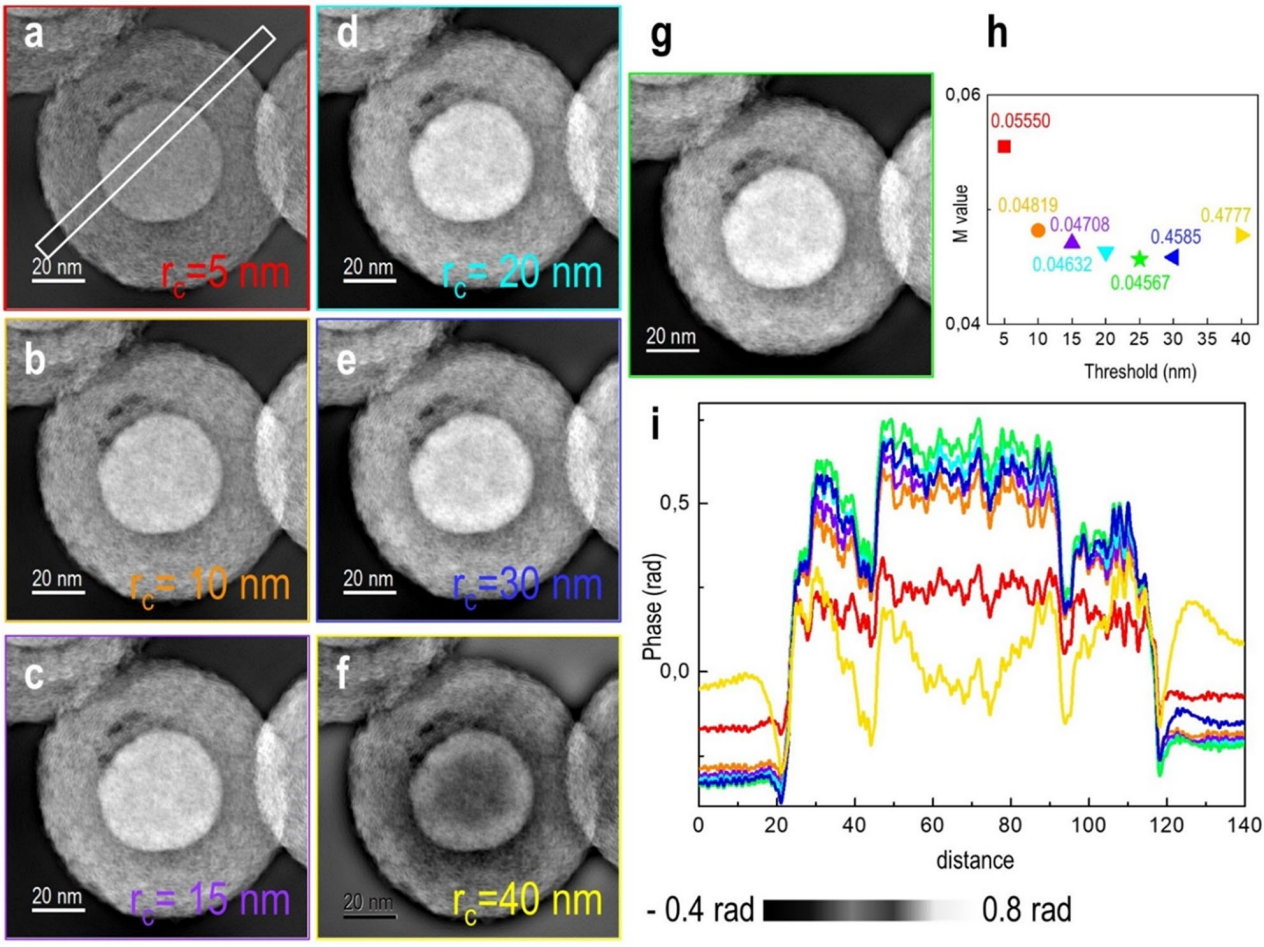
Phase images of Fe-filled C nanospheres reconstructed using (**a**) 5, (**b**) 10, (**c**) 15, (**d**) 20, (**e**) 30, (**f**) 40, and (**g**) 25 nm threshold values. (**h**) Threshold vs. M value (mismatch between simulated and experimental images). (**i**) Line profiles of (**a**–(**g**) from the region marked in (**a**).

We now discuss the effect of only the parameter *r*_c_ in Eq. (3). This parameter determines the real space length scale above which gradient flipping becomes effective. Phase information for distances below *r*_c_ is determined by the iterative FRWR reconstruction algorithm, with GF only affecting relative phases across greater distances, where the phase prediction is off. Figure 2 shows phase images and profiles reconstructed using different values of *r*_c_ for the Fe-filled C nanospheres. Increasing *r*_c_ initially improves the contrast in the phase. Figure 2a–d shows that the lowest contrast is obtained for *r*_c_ = 5 nm. The highest contrast is obtained with *r*_c_ set to 25 nm (Fig. 2g). Increasing *r*_c_ further does not improve the contrast.

If *r*_*c*_ is set to be much higher than the lateral coherence length of the incident electron wave function, then the reconstruction proceeds as if no gradient flipping had been applied, since the exponential in Eq. (3) only includes very low spatial frequencies. For this reason, dark ringing features appear around the particle in Fig. 2f when the threshold value is too high.

The *M* value defined in Eq. (2), which measures the mismatch between experimental and simulated images during reconstruction, is also smallest at *r*_c_ = 25 nm (Fig. 2g). Figure 2h shows the dependence of *M* on *r*_c_. The minimum value is obtained when the contrast is maximized at *r*_*c*_ = 25 nm, indicating that *M* is likely to be a suitable figure of merit for optimizing *r*_*c*_.

The profile taken from the GFRWR reconstruction shows an offset in phase between vacuum regions on opposite sides of the core–shell particle (Fig. 2i). This problem arises because the vacuum regions are not connected. Ideally, both sides of the particle should have the same phase shift. We again observe minimum phase offset differences for minimum *M* and highest contrast, i.e., where r_c_ is 25 nm.

**Figure 3:**
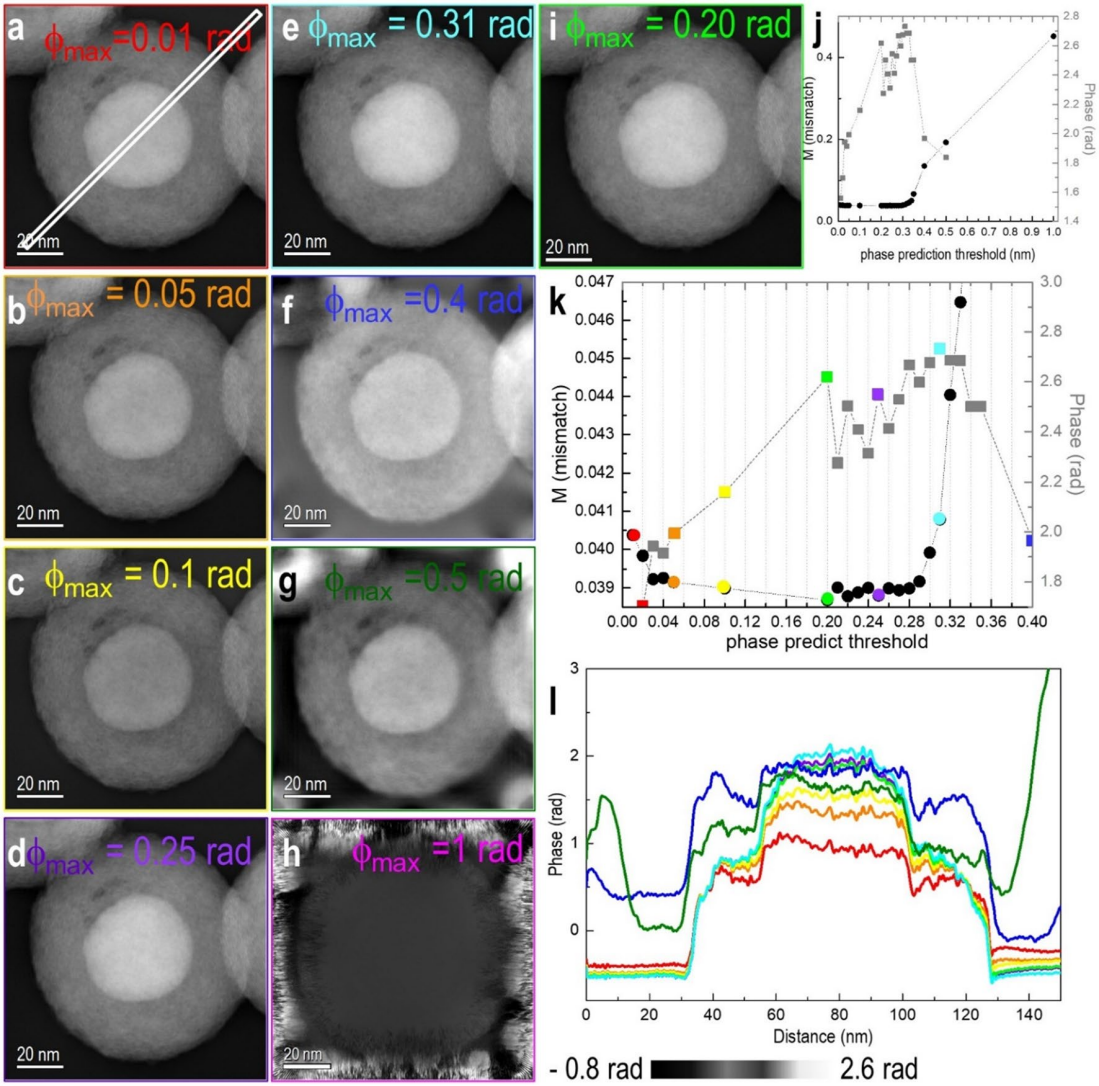
Phase images of Fe-filled C nanospheres reconstructed using phase prediction thresholds of (**a**) 0.01, (**b**) 0.05, (**c**) 0.1, (**d**) 0.25, (**e**) 0.31, (**f**) 0.4, (**g**) 0.5, (**h**) 1.0 and (**i**) 0.20 rad. (**j**) Phase prediction threshold vs M value (mismatch between simulated and experimental images) and phase contrast. (**k**) Magnified version of (**j**). (**l**) Line profiles extracted from the region marked in a) for phase images (**a**) to (**i**). The grey squares represent phase, and black circles are mismatch values M in (**j**) and (**k**) and colors represent the *ϕ*_max_ that is presented with the images.

Figure 3 shows changes in the reconstructed phase as a function of only phase prediction threshold (PPT), PFRWR. (See the “Theory” section for details). A similar procedure to that used for optimizing *r*_*c*_ was followed to find the best condition for a TIE-like phase prediction. Reconstructions were performed for PPT *ϕ*_max_ = 0.01 rad and above. A shallow minimum was found for PPT *ϕ*_max_ = 0.2 rad.

When *ϕ*_max_ was set to 0.4 or 0.5 rad (Fig. 3f,g), reconstruction artifacts became obvious. For the more extreme case of *ϕ*_max_ = 1 rad, the phase could no longer be reconstructed (Fig. 3h). For both this dataset and *ϕ*_max_ = 0.31 rad, the reconstruction diverged when the number of iterations exceeded 2500. For *ϕ*_max_ = 0.20 rad, the algorithm diverged when it was run for more than 4000 iterations. This behavior seems to be related to the way in which the phase is treated in the padded area outside the field of view, where experimental intensity data are available. Since it is not practical to use such large numbers of iterations, this is not a limitation for the application of the algorithm. After the determination of the ideal values for *r*_*c*_ and *ϕ*_max_ using a small number of iterations, the iteration number was increased to obtain a minimum value of the mismatch *M*, which was closest to the actual object wave. The reconstruction was started with 50 iterations to find roughly optimum values for *ϕ*_max_ and r_c_, and then the iteration number was increased up to 1000 for further refinement of the phase until the M values converged for the Fe core-shell C particle reconstruction.

**Figure 4:**
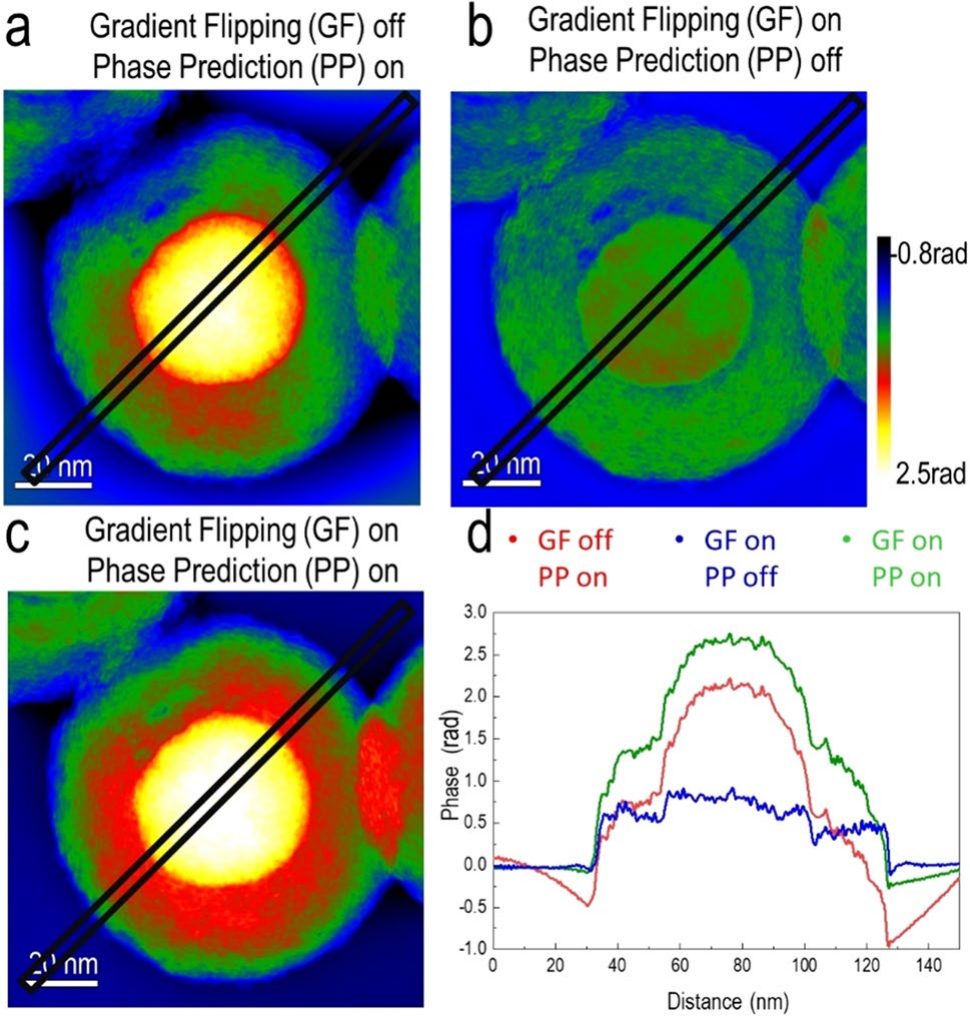
Comparison between individual and combined effects of gradient flipping and phase prediction. (**a**) Phase reconstruction with gradient flipping (GF) off and phase prediction (PP) on. (**b**) Phase reconstruction with GF on and PP off. (**c**) Phase reconstruction with GF on and PP on. (**d**) Line profiles extracted along the lines marked in the phase maps.

The individual and combined effects of phase prediction and gradient flipping are shown in Fig. 4. The comparison shows that phase prediction increases the contrast significantly and can recover much lower spatial frequencies in the phase than the iterative non-linear flux-preserving focal series reconstruction algorithm alone, even when gradient flipping is turned on. Figure 4a and the red line profile in Fig. 4d show that phase prediction results in extended contrast. The same figure shows significant edge artifacts associated with missing low spatial frequencies when gradient flipping is not used. Gradient flipping corrects for the missing low spatial frequency information by finding a phase image that is consistent with the experimental data and, at the same time, sparse in its gradient. The blue profile in Fig. 4d shows a constant phase profile in the vacuum region, where no phase variations are expected. Although both gradient flipping and phase prediction individually have strong effects on the reconstructed phase, the most significant improvement results from the use of gradient flipping and phase prediction together. Figure 4c and the green profile in Fig. 4d show how edge artifacts are then significantly reduced.

## Conclusions

We have shown that a charge flipping algorithm, which is successful in solving the crystallographic phase problem in diffraction for finding solutions that are sparse in the space of charge density (X-ray diffraction) or electrostatic potential (electron diffraction), can be adapted to recover phase images that are sparse in their gradient domain using a non-linear flux-preserving inline holography reconstruction algorithm. A non-linear iterative gradient flipping reconstruction algorithm has been developed that overcomes the limitations of conventional reconstruction algorithms due to both an incoherent background in the image intensities and poor encoding of low spatial frequency phase information. Its ability to recover low spatial frequency phase information is demonstrated for two experimental test cases, and the results are compared with off-axis electron holography. For these test cases, the combination of gradient flipping with phase prediction, incoherent background subtraction, and a non-linear flux-preserving iterative reconstruction scheme provide semi-quantitative phase maps that recover approximately 80% of the phase contrast reconstructed using off-axis electron holography.

## Supplementary Information

Supplementary Information.

## Data Availability

The datasets used and/or analyzed during the current study are available from the corresponding author on reasonable request.
