# Supplementary material for: Synergistic use of gradient flipping and phase prediction for inline electron holography
Source: Sci Rep. 2022 Aug 2;12:13294. doi: 10.1038/s41598-022-17373-y (PMC9345894; doi:10.1038/s41598-022-17373-y)
Supplement: Supplementary file 1 — Supplementary Information. [file 41598_2022_17373_MOESM1_ESM.docx]

**Synergistic use of Gradient Flipping and Phase Prediction**

**for Inline Electron Holograph**

Cigdem Ozsoy-Keskinbora^1*†⸸^, Wouter Van den Broek^2†^,
 Chris B. Boothroyd^3,4^, Rafal E. Dunin-Borkowski^3^, Peter A. van Aken^1^,
 Christoph T. Koch^2**^

^1^ Stuttgart Center for Electron Microscopy, Max Planck Institute for Solid State Research,

Heisenbergstr. 1, 70569 Stuttgart, Germany

^2^ Department Physics & IRIS Adlershof, Humboldt-Universität zu Berlin, 12489 Berlin, Germany

^3^ Ernst Ruska-Centre for Microscopy and Spectroscopy with Electrons and Peter Grünberg Institute,

Forschungszentrum Jülich, 52425 Jülich, Germany

^4^School of Materials Science and Engineering, Nanyang Technological University, Singapore

Subject area: Optics, holography, transmission electron microscopy, iterative reconstruction, phase-contrast imaging

* cigdem.ozsoykeskinbora@thermofisher.com

** christoph.koch@hu-berlin.de

^†^ Present address: Thermo Fisher Scientific, Eindhoven, The Netherlands

^⸸^ Present address: Harvard University, Cambridge, USA

**SUPPLEMENTARY INFORMATION**

Gradient-flipping and Phase prediction-assisted Flux preserving- full-resolution wave reconstruction (GPFRWR), off-axis, and in-line electron holography experiments were carried out for two different samples. MgO cubes (Fig. 1a) and core-shell Fe-filled C nanospheres (Fig. 1b) were selected as test specimens. MgO cubes have previously been used in electron holography studies^1^. The MgO cubes have sharp edges, while the core-shell particles have fine features with sizes of between ~0.5 and 0.8 nm, thereby allowing the applicability of the method to be assessed for a wide range of spatial frequencies.


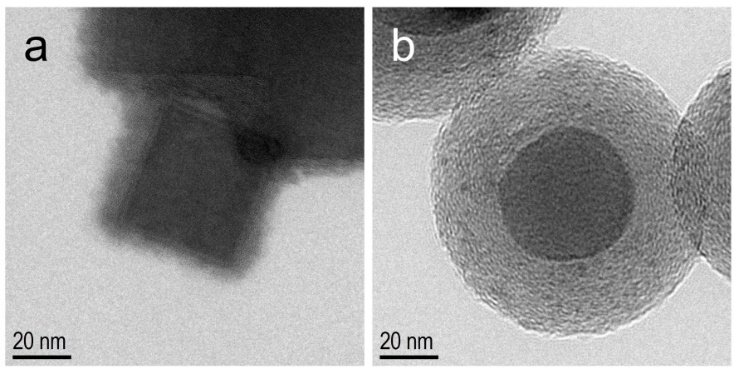


FIG. 1. Bright-field TEM images of a) MgO cubes covered by some amorphous carbon; b) Fe-filled C nanospheres.

Figure 2 shows phase images of MgO cubes obtained using the conventional iterative flux-preserving in-line holography reconstruction algorithm^2^ (Fig. 2a), the same flux-preserving approximation with gradient flipping applied (Fig. 2b) and off-axis holography (Fig. 2 c). The phase obtained using the conventional in-line reconstruction algorithm varies over the range -2 to 6 rad, which is ~ 50% lower than that recovered using off-axis holography. The rapid decrease in phase for iterative reconstruction just outside the MgO cube (Fig. 2a and the red profile in Fig. 2d) results from missing low spatial frequencies. Figure 2b and the corresponding line scan in Fig. 2d show that gradient flipping prevents artifacts at the edges of the MgO cube and results in a homogeneous background. The agreement between Figs 2b and 2c is satisfying. The remaining difference of ~20% between the GPFWR and off-axis results may be attributed partially to imperfect energy filtering during the acquisition of the focal series. It should be noted that off-axis electron holography inherently filters out inelastic scattering^3,4^, whereas in-line electron holography has to rely on the use of an energy filter. In order to reduce the influence of inelastic scattering, the imaging model used here calculates and subtracts the inelastic contribution to first order.

The power spectra are shown in Fig. 2e highlight the differences in information transfer between the three methods. The phase resolutions obtained using in-line and off-axis electron holography are 0.34 nm and 1.2 nm, respectively. (The fields of view are identical, but a reciprocal space mask was applied during the reconstruction of the wave function from the off-axis data in order to separate center- and side bands and to achieve a reasonable signal to noise ratio). The power spectra show that the GPFRWR algorithm recovers information reliably over distances of up to ~80 nm, whereas the FRWR reconstruction is only reliable over distances of up to ~30 nm.

Although the GPFRWR algorithm did not recover phase differences reliably over distances larger than ~80 nm, the present example showed an improvement over the conventional reconstruction algorithm, with dramatically reduced artifacts at the edges of the particles originating from missing low spatial frequencies. The reconstructed phase images look much closer to results obtained using off-axis holography while retaining the superior spatial resolution of focal series reconstruction. This approach may help in the measurement of quantities such as variations in mean inner potential across short distances.


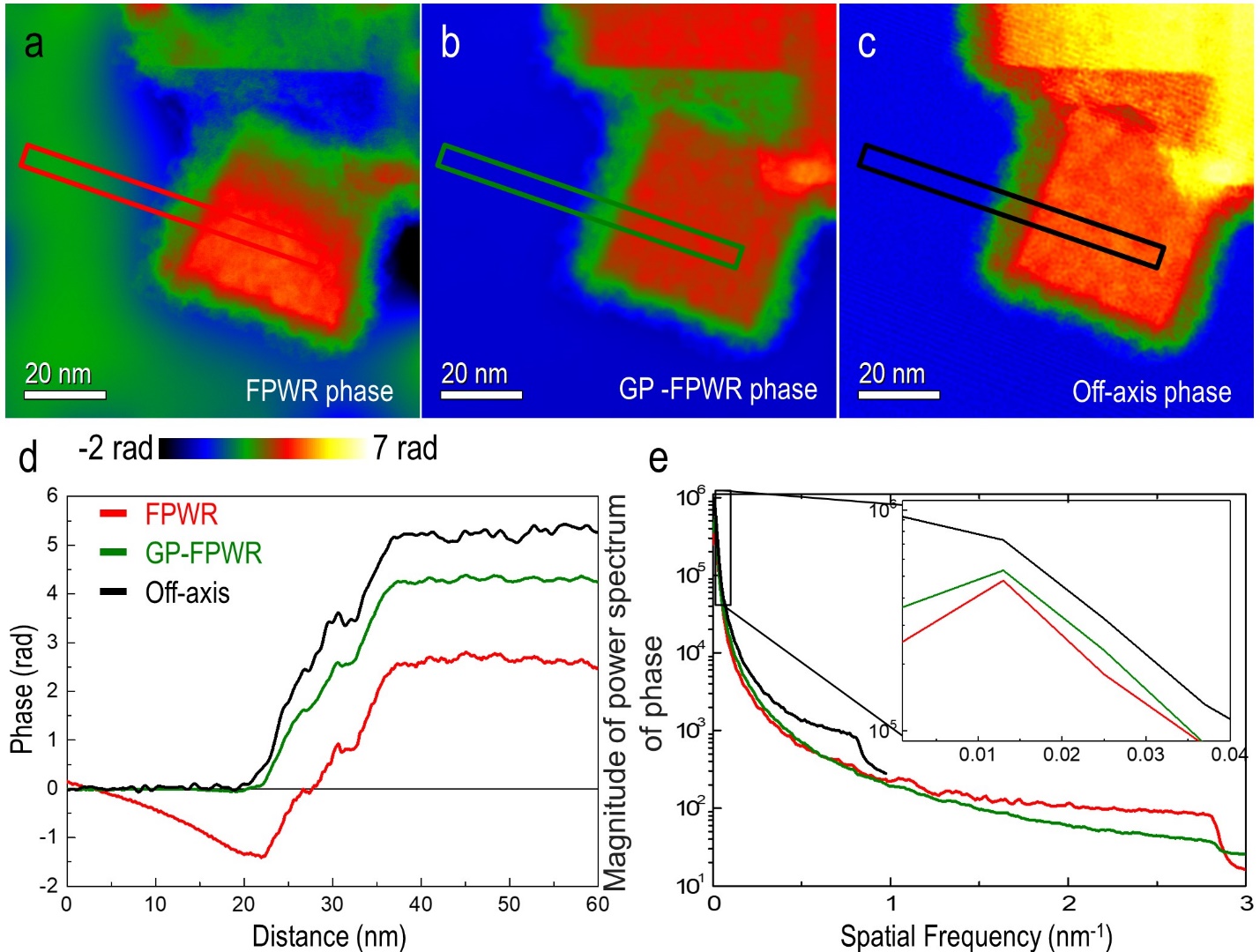


FIG. 2. Phase images of MgO cubes reconstructed using a) FRWR, b) GPFRWR, and c) off-axis electron holography. d) Line profiles extracted from the three phase reconstructions. e) Radially-averaged power spectra of the three phase images.

References

1. Gatel, C., Lubk, A., Pozzi, G., Snoeck, E. & Hÿtch, M. Counting Elementary Charges on Nanoparticles by Electron Holography. *Phys. Rev. Lett.* **111**, 025501 (2013).

2. Koch, C. T. A flux-preserving non-linear inline holography reconstruction algorithm for partially coherent electrons. *Ultramicroscopy* **108**, 141–150 (2007).

3. Lichte, H., Geiger, D. & Linck, M. Off-axis electron holography in an aberration-corrected transmission electron microscope. *Philos. Trans. R. Soc. Math. Phys. Eng. Sci.* **367**, 3773–3793 (2009).

4. Ozsoy-Keskinbora, C., Boothroyd, C. B., Dunin-Borkowski, R. E., van Aken, P. A. & Koch, C. T. Mapping the electrostatic potential of Au nanoparticles using hybrid electron holography. *Ultramicroscopy* **165**, 8–14 (2016).
